# Two Generations of Mother-Child Relationships: A Self-determination Theory Analysis of the Social Situation of Development

**DOI:** 10.11621/pir.2021.0410

**Published:** 2021-05-31

**Authors:** Nailya R. Salikhova, Martin F. Lynch, Albina B. Salikhova

**Affiliations:** a Kazan Federal University, Kazan, Russia; b University of Rochester, Rochester, New York, USA; c National Research University Higher School of Economics, Moscow, Russia; d I.M. Sechenov First Moscow State Medical University (Sechenov University), Moscow, Russia

**Keywords:** Social situation of development, parent-child interaction, intergenerational continuity, basic psychological needs, basic need support, self-determination theory (SDT)

## Abstract

**Background:**

This study used self-determination theory to examine the intergenerational continuity of the social situation of development with a focus on what determines a woman’s basic psychological need support for her child.

**Objective:**

To assess the relationship between the basic need support a woman received from her own mother, the woman’s basic need support toward her own child, and the quality of the woman-child interaction.

**Design:**

The scales, “Parent-child interaction” and “Basic Psychological Needs,” were administered. Eighty-seven women (29-40 years old) with children age 4-5 years assessed the basic need support provided for them by their mother in childhood and at present, and her estimate of the basic need support she herself provides to her own child. Analyses included descriptive statistics, Wilcoxon signed-rank tests, factor analysis, and multiple linear regression.

**Results:**

The ratio of levels of basic need support demonstrated continuity across generations. Intergenerational continuity in the child’s basic need support mainly concerns the needs for competence and relatedness: the more they were supported in childhood and are now supported by the woman’s mother, the more the woman supports them in her own child today. Such continuity was not found for autonomy support. A woman’s own basic need support by her mother, in childhood and currently, and the woman’s provision of basic need support for her child predicted most of the woman-child interaction parameters.

**Conclusion:**

Intergenerational continuity with respect to provision of basic need support was shown. The woman-child interaction was predicted by basic need support across intergenerational relations.

## Introduction

### Research problem

The concept of the “social situation of development” is one of the central concepts pertaining to age-related development in the Cultural-Historical theory of L.S. Vygotsky (1984). It has become an alternative to the concept of “environment” and denotes the entire completeness and uniqueness of the child’s relationship with the world at every stage of their development. The structure of the social situation of development includes such components as the child’s place in the system of relationships, the attitude of the environment towards the child, the attitude of the child to those around them, and their attitude toward their place in the relationship system. Despite the theoretical, heuristic nature of this concept, further development of the methods of its theoretical and empirical analysis is necessary.

In psychology, the attitude of the parents to the child, as a component of the social situation of development, is most actively investigated (Smirnova, 2017; Sobkin et al., 2016); the attitude of the child to the parents is somewhat less studied (Markovskaya, 2007). At the same time, research results are rarely generalized to characterize a holistic social developmental situation. Among the determinants of parental attitudes towards a child, the most often considered are the individual characteristics of the parents, and the individual characteristics of the child, including the child’s state of health.

Self-determination theory offers a framework for analyzing the social situation of development by exploring parental support for the child’s basic psychological needs, or, more briefly, basic needs. There has been substantial empirical support for the claims of self-determination theory (SDT) regarding the importance, for the child’s psychological well-being and healthy development, of support by the proximal environment for the child’s basic psychological needs for autonomy, competence, and relatedness (Ryan & Deci, 2017; Vasquez, Patall, Fong, Corrigan, & Pine, 2016). A look at the social situation of development through the prism of basic psychological needs and their support provides a rich portrait for analysis. In turn, research on intergenerational continuity can be an effective way to uncover the factors that influence the ability of adults to provide basic need support to the child, with respect to the needs for autonomy, competence, and relatedness. The Vygotskian notion of the social situation of development makes it possible to substantiate, theoretically, the existence of such continuity. The child, interacting with the environment, masters not only symbolic means as internal tools of mental functions, but also internalizes the entire drama of relationships in which these means were included (Vygotsky, 1983).

The key role of the mother-child relationship in the formation of the child’s personality is widely known. At the same time, little is known about what exactly determines a woman’s ability to provide basic need support for her child. Perhaps this support depends on the extent to which the woman’s own mother supported her basic psychological needs during childhood, and the extent to which she continues to do so today. Psychologists have shown the transferability of parental behavior patterns from generation to generation (Bailey, Hill, Oesterle, & Hawkins, 2009), although the main focus has been on the transmission of maladaptive parenting strategies (Seay, Jahromi, Umaña-Taylor, & Updegraff, 2016). We assume that positive basic need support for a child’s needs for autonomy, competence, and relatedness can also be passed on from generation to generation, specifically, that the basic need support a woman received and currently receives from her own mother, has an influence on her interactions with her child. Until now, there have been no studies in Russia, based on SDT, on the continuity or intergenerational transmission of basic need support towards a child.

In psychology, ever since the work of Freud, much attention has been paid to the attitude of parents toward their children. It is known that the relationship between parent and child greatly influences many characteristics of the child. In particular, much attention has been paid to the study of negative and traumatic influences. Such studies do not lose their relevance at the present time. Researchers have shown intergenerational continuity in antisocial behavior (Thornberry, Freeman-Gallant, Lizotte, Krohn, & Smith, 2003) and in the use of psychoactive substances (Bailey et al., 2009). Yet, some scientists have shown that the influence of parents on child development is not as unambiguous as earlier researchers assumed, but is also not as insignificant as modern critics argue (Collins, Maccoby, Steinberg, Hetherington, & Bornstein, 2000).

### Continuity of generations in the way a parent interacts with a child

An earlier generation, consciously or unintentionally, psychologically influences the parenting attitudes and behavior of the next generation (Van Ijzendoorn, 1992). For example, there is a significant correlation between mothers and grandmothers in the use of physical punishment and material rewards (Covell, Grusec, & King, 1995).

Studies on the transmission of negative parenting practices such as harsh parenting (Simons, Whitbeck, Conger, & Chyi-In, 1991), aggressive and hostile parenting (Conger, Neppl, Kim, & Scaramella, 2003; Hops, Davis, Leve, & Sheeber, 2003; Scaramella & Conger, 2003), child abuse (Seay et al., 2016), tight parental control and discipline (Bailey et al., 2009), and conditional regard (Assor, Roth, & Deci, 2004), have shown that poor parenting practices are transmitted through generations of parents and children, and that this has a mediated effect through the development of antisocial and delinquent behavior in the child by the time of adolescence (Capaldi, Pears, Patterson, & Owen, 2003). Even the high quality of the current relationship between caregivers does not eliminate such negative effects (Belsky, Youngblade, & Pensky, 1989).

The emphasis on the study of the transmission from generation to generation of destructive models of parental attitude to the child, naturally led to the question of how this also applies to positive models of behavior. Indeed, research has shown the intergenerational continuity of constructive parenting (Chen & Kaplan, 2001). Not only has the continuity of a positive relationship been investigated, but also its mechanisms (Neppl, Conger, Scaramella, & Ontai, 2009; Prokhorov, Chernov, & Yusupov, 2015), and the role of other circumstances and mediators in explaining continuity and influencing the magnitude of its effect between generations (Belsky, Sligo, Jaffee, Woodward, & Silva, 2005; Schofield, Conger, & Neppl, 2014).

### Parental support for the basic needs of the child and the child’s well-being

Parental basic need support is essential for the child’s effective development and well-being. In self-determination theory (SDT), there is substantial evidence of this for children of different ages. Supporting a child’s basic needs has a positive effect on the child’s psychological health, academic performance in school, behavior, etc. When basic need support (from the parent) leads to basic need satisfaction (in the child), this need satisfaction mediates between the child’s perception of psychological control and the internalization of distress. Moreover, parental psychological control has been found to be a better predictor of distress internalization than low parental autonomy support (Costa, Soenens, Gugliandolo, Cuzzocrea, & Larcan, 2015).

SDT pays special attention to supporting the need for autonomy. Moreover, it is important to distinguish support for autonomy, from the promotion of permissiveness or independence (Ryan & Deci, 2017). Many authors present studies that examine how parental support for autonomy, as one of the basic needs recognized in SDT, contributes to the healthy development of children of different ages (Joussemet, Landry, & Koestner, 2008). Maternal support for autonomy contributes to the development of autobiographical narrative skills in children (Leyva, Reese, Grolnick, & Price, 2008). When parents are perceived by their children as autonomy-supportive, children more highly rate their satisfaction of talking to the parent about shared memories (Van der Kaap-Deeder, Soenens, Mouratidis, De Pauw, Krøjgaard, & Vansteenkiste, 2020). Support for the adolescent’s autonomy by her mother predicts increased emotional integration and decreased suppressive regulation in adolescents (Brenning, Soenens, Petegem, & Vansteenkiste, 2015).

SDT is also used in intervention and corrective practices to improve the effectiveness of parenting, by helping parents to be more consistent in providing basic need support for their children. Several studies show that SDT-based intervention programs are effective (Allen, Grolnick, & Córdova 2019). By helping parents to support the child’s psychological needs for autonomy, competence, and relatedness, such programs help increase the child’s autonomous self-regulation (i.e., their ability to internally self-regulate), and consequently reduce behavior problems in children (Grolnick, Levitt, Caruso, & Lerner, 2021).

### Cross-cultural differences in parental basic need support

Cross-cultural research confirms the universality of basic psychological needs and the positive impact of their satisfaction on the well-being and healthy development of the child (Chen et al., 2015; Ryan & Deci, 2017). For example, a study conducted in Japan found that when fathers had relatively high goals in life, both fathers and their teenage children experienced greater satisfaction of basic psychological needs (Nishimura, Bradshaw, Deci, & Ryan, 2021). Supporting basic psychological needs affected the life aspirations and psychological well-being of South African 16 year-old students (Roman et al., 2015).

At the same time, researchers have also identified some cultural differences. For example, supporting the need for autonomy has been associated with positive outcomes among adolescents, but this link in collectivist cultures is unclear, as manifested when comparing adolescents from the United States and Ghana (Marbell-Pierre, Grolnick, Stewart, & Raftery-Helmer, 2019). In a survey of Russian subject-matter teachers, kindergarten teachers, pedagogical psychologists, and managers of education departments, the need for autonomy was valued less compared to the needs for relatedness and competence (Lynch & Salikhova, 2016; see also Lynch, in press).

### Generational continuity in basic need support by the parent

Little research has been done on intergenerational continuity in the provision of basic need support, but there is evidence that it exists. Costa et al. (2019) showed that parents who have experienced high levels of basic need support from their own parents tend to provide more support for the need for autonomy in their relationships with their children. Conversely, if parents experienced a high level of frustration of their own basic psychological needs during childhood, then they were more likely to use psychological control and cause feelings of need frustration in their children (Costa, Gugliandolo, Barberis, Cuzzocrea, & Liga, 2019). The mother’s perception of parental experience (in the family of origin) has been shown to be associated with the self-reported use of psychological control by mothers during early childhood (Brenning, Soenens, Van der Kaap-Deeder, Dieleman, & Vansteenkiste, 2020), and there is evidence of generational transmission of parental conditional regard, as well (Assor et al., 2004). We note here that control and conditional regard are considered, from the perspective of SDT, to be parenting strategies that are antithetical to support for the child’s basic needs, in particular, the need for autonomy.

### Purpose and hypotheses of the current study

The aim of the study was to reveal whether there is intergenerational continuity of the social situation of development with respect to basic need support for the child. Based on self-determination theory, we made the following predictions:

Hypothesis 1 (H1): The level of basic need support that a woman receives from her mother, and that she provides to her own child in the present, are similar.

Hypothesis 2 (H2): A woman who herself experienced basic need support from her mother, both in childhood and at present, will be more likely to provide basic need support to her own child.

Hypothesis 3 (H3): The nature of the woman’s interaction with her child in the present will be predicted by the child’s basic need support across intergenerational systems.

## Methods

### Participants

This study involved 87 women 29–40 years old (M = 34.7, SD = 3.1). All women live in Nizhny Novgorod (Russia) and belong to the ethnic majority (Russians); 82% had higher education, and 18% had specialized secondary education. The majority (93%) had a job; 7% indicated that they were housewives. Each woman had a child age 4–5 and her own still-living mother, with whom the woman was currently interacting.

### Measures

#### “Basic Psychological Needs Scales” (La Guardia, Ryan, Couchman, & Deci, 2000)

This measure contains nine questions that together determine to what extent, in the context of a particular interaction, a person perceives their need for support for: autonomy (3items, α’s ranging from .59 to .71); competence (3items, α’s ranging from .61 to .78); and relatedness (3items, α’s ranging from .64 to .76). Items were scored on a scale of 1 (completely inaccurate) to 7 (completely accurate). A high cumulative score on each scale indicates greater support for the corresponding need. Sample items include: “When I am with my mother, I feel free to be who I am” (autonomy); “When I am with my mother, I feel very capable and effective” (competence); and “When I am with my mother, I feel a lot of closeness and intimacy” (relatedness).

#### “Parent-child interaction” (Markovskaya, 2005)

This measure assesses 10 parameters of a woman’s interaction with her child: “undemanding — exactingness” (5 items, Cronbach’s α = .69); “softness — strictness” (5items, Cronbach’s α = .67); “autonomy — control in relation to the child” (10items, Cronbach’s α = .63); “emotional distance — emotional closeness of the child to the parent” (5items, Cronbach’s α = .72); “rejection — acceptance of the child by the parent” (10 items, Cronbach’s α = .77); “lack of cooperation — cooperation” (5 items, Cronbach’s α = .74); “disagreement — agreement” (5 items, Cronbach’s α = .61); “inconsistency — consistency” (5 items, Cronbach’s α = .63); “parental authority” (5items, Cronbach’s α = .80); and “satisfaction with the relationship” (5items, Cronbach’s α = .71). Items were scored on a scale of 1 (no, absolute disagreement) to 5 (undoubtedly yes, very strong agreement). Higher scores on the first eight scales correspond to the right pole of these scales and, for the last two scales, to a greater extremity with respect to the measured parameters.

### Procedure

This study was conducted in a kindergarten. The women took part in the study because they were interested in receiving feedback from a psychologist. Each participant was provided with the results of their own scale scores, and the interpretation of the results was explained to them during a specially-organized lecture.

Participation was voluntary, with no compensation, and anonymity was guaranteed. All participants gave verbal consent to the use of their data in anonymized form for the purposes of scientific research. All measures were taken at the same time.

Each woman who participated in the study responded to items on the “Basic Psychological Needs Scales” scale three times, with respect to three different relationship contexts. Specifically, we obtained three indicators of support for autonomy, competence, and relatedness, once for each of three different relationship systems. First, each woman assessed how her mother supported her basic needs in her childhood (Woman–Her Mother–Childhood system, abbreviated W_HM_Ch), yielding indicators of support for autonomy (A_W_HM_Ch), competence (C_W_HM_Ch), and relatedness (R_W_HM_Ch). Second, each woman assessed how her mother was providing basic need support to her at the present time (Woman–Her Mother–Nowsystem, or abbreviated W_HM_Now), which yielded indicators of support for autonomy, (A_W_HM_Now); competence (C_W_HM_Now); and relatedness (R_W_HM_Now). Thirdly, she answered from the perspective of her child and assessed how she herself provided basic need support to the child, from the point of view of her child (Woman–Baby–Now system, or abbreviated W_B_Now), and these yielded indicators of support for the child’s autonomy (A_W_B_Now); competence (C_W_B_Now); and relatedness (R_W_B_Now). Then each woman responded to items on the “Parent-child interaction” scale.

### Data analysis

First, a factor analysis of basic need support in all studied relationship systems (W_ HM_Ch, W_HM_Now and W_B_Now systems) was carried out. The purpose was to analyze whether a woman, answering the same questions for three different systems of relationships at the same time, distinguished these systems from each other.

Then, using descriptive statistics and the two-samples paired Wilcoxon test, we compared, first, basic need supports within each of the relationship systems, and secondly, basic need support across different relationship systems, in order to test Hypothesis 1 (H1).

Finally, we used multiple linear regression to examine, first, the extent to which basic need support by her mother now and in childhood was a predictor of a woman’s support for the basic psychological needs of her child (H2); and secondly, to what extent basic need support in all studied systems of relations was a predictor of the parameters of child-parent interaction between a woman and her child (H3). Before carrying out multiple linear regression, using the curve estimation procedure, functions were determined that optimally approximated the relationship between the independent and dependent variables of the models.

## Results

The results of factor analysis are presented in *Tables 1* and *2*. Principal components analysis (rotation method: direct oblimin) makes it possible to assert greater similarity between variables within one system of relationships than between systems: three automatically-generated factors coincided in their content with the three systems of relationships we are considering (see *Table 1*). Moreover, the scores of the basic need support items in the relationship of a woman with her mother now and in childhood, according to the interfactor correlation matrix, turned out to be more similar than they were with the child’s need support scores (see *Table 2*). This suggests that the participants, when completing items with respect to the various targets, clearly distinguished the systems of relationships they assessed.

**Table 1:** Factor loading matrix of basic need support in various systems of relationships (method of principal components, rotation method — direct oblimin with Kaiser normalization)

	Factors		
	Factor 1	Factor 2	Factor 3
	(W_HM_Now)	(W_B_Now)	(W_HM_Ch)
C_W_HM_Now	**0.900**	0.065	0.000
A_W_HM_Now	**0.880**	–0.070	0.052
R_W_HM_Now	**0.650**	0.083	–0.378
A_W_B_Now	0.058	**0.878**	0.190
C_W_B_Now	–0.223	**0.844**	–0.166
R_W_B_Now	0.301	**0.651**	–0.144
A_W_HM_Ch	–0.139	–0.061	**–0.940**
C_W_HM_Ch	0.071	0.113	**–0.847**
R_W_HM_Ch	0.297	0.015	**–0.699**
Percentage of variance	44.7%	17.3%	14.4%

*Note. Factor solution explains 76.4 % of variance. The factor loadings that are most closely related to components are highlighted in bold*.

**Table 2:** Correlation matrix of components

	Factor 2 (W_B_Now)	Factor 3 (W_HM_Ch)
Factor 1 (W_HM_Now)	0.200	-0.326**
Factor 2 (W_B_Now)		-0.265*

Note. * p<0.05; ** p<0.01.

Comparison of measures of basic need support are presented in *Table 3*.

**Table 3:** Comparison of measures of basic need support

System of relation- ships	Autonomy MSD	Competence M SD	Relatedness M SD	Friedman X^2^	test p	Comparison of needs support	Wilcoxon Z	test p
W_HM_Ch	15.05	16.45	16.70	32.829	0.000	A / C	–5.037	0.000
	2.799	2.782	3.328			A / R	–4.619	0.000
						C / R	–1.343	0.179
W_HM_Now	19.03	19.01	18.38	3.801	0.150	A / C	–0.058	0.953
	1.877	2.414	3.100			A / R	–1.930	0.054
						C / R	–2.348	0.019
W_B_Now	17.94	19.16	19.47	55.565	0.000	A / C	–5.704	0.000
	2.253	1.516	2.188			A / R	–5.193	0.000
						C / R	–1.624	0.104

*Note. M — mean score; SD — standard deviation; X^2^ — Pearson’s chi-squared test; Z — Wilcoxon Rank-Sum test statistic; p — significance*.

As we can see, support for autonomy was lower than all other needs, while support for relatedness and competence were not significantly different in the W_HM_ Ch and W_B_Now systems of relations.

Comparison of measures of basic need support between different systems of relationships is presented in *Table 4*.

**Table 4:** Comparison of measures of basic need support between di% erent systems of relationships

Support of needs	W_HM_Ch Mean	W_HM_Now Mean	W_B_Now Mean	Friedman test		Comparison of relationships s ystems	Wilcoxon test	
	SD	SD	SD	X^2^	P		Z	P
Autonomy	15.05	19.03	17.94	78.426	0.000	W_HM_Ch / W_HM_Now	–7.602	0.000
	2.799	1.877	2.253			W_HM_Ch / W_B_Now	–6.155	0.000
						W_HM_Now / W_B_Now	–4.015	0.000
Competence	16.45	19.01	19.16	52.234	0.000	W_HM_Ch / W_HM_Now	–6.163	0.000
	2.782	2.414	1.516			W_HM_Ch / W_B_Now	–6.765	0.000
						W_HM_Now / W_B_Now	–0.071	0.943
Relatedness	16.70	18.38	19.47	55.063	0.000	W_HM_Ch / W_HM_Now	–5.014	0.000
	3.328	3.100	2.188			W_HM_Ch / W_B_Now	–6.473	0.000
						W_HM_Now / W_B_Now	–3.421	0.001

*Note. M— mean score; SD— standard deviation; X^2^— Pearson’s chi-squared test; Z— Wilcoxon Rank-Sum test statistic; p— significance.*

It can be seen that women rated their mother’s current basic need support higher, compared to her basic need support in childhood.

The woman herself believed that she currently supports the basic needs of her child more than her mother supported her basic needs in her childhood.

A preliminary assessment of the curvilinear relationship between basic need support of a child by their mother, and of a woman by her mother in childhood and now, as well as between basic need support in the studied systems of relationships and interaction (Markovskaya, 2005) in the mother-child system, showed that in 90% of the relationships between the dependent and independent variables, quadratic models explained more variance in the dependent variable than linear models. In the remaining 10% of the relationships, the linear and quadratic models explained the same percentage of variance in the dependent variable. Therefore, in the subsequent analysis, both basic need support scores and squares of these scores were included in the regression models as independent variables. The results of regression analysis in relation to the variables of a woman’s basic need support for her child and the parameters of parent-child relationships, for which significant predictors were identified, are presented in *Tables 5* and *6*.

**Table 5:** Multiple linear regression (stepwise selection method) of the dependence of basic need support of a child in relationships with their mother (according to mother’s assessment) on the woman’s basic need support in relationships with her mother in her childhood and at the present time

Dependent variable: basic need support	R^2^	Durbin– Watson statistic	ANOVA		Independent variables: basic need support	Coefficients		
			F	p		B	SE	β
A_W_B_Now	0.059	1.897	5.327	0.023	Constant	14.698	1.425	
					R_W_HM_Now	0.177	0.076	0.243
C_W_B_Now	0.099	2.148	9.375	0.003	Constant	17.716	0.497	
					C_W_HM_Ch (square)	0.005	0.002	0.315
R_W_B_Now	0.458	2.029	17.326	0.000	Constant	–9.395	4.644	
					R_W_HM_Ch	2.818	0.563	4.285
					R_W_HM_Ch (square)	–0.085	0.018	–4.134
					C_W_HM_Ch	0.281	0.088	0.357
					C_W_HM_Now (square)	0.005	0.002	0.204

Note. B— non-standardized coefficient of the regression equation; SE is the standard error; β is the standardized coefficient of the regression equation; R2 is the coefficient of determination; F = Fisher’s F-test; p = significance. Critical probability = 5 %.

**Table 6:** Multiple linear regression (stepwise selection method) of the dependence of a woman’s quality of interaction with her child on the woman’s basic need support in relationships with her mother in her childhood, at the present time, and the basic need support of her child in relationships with the woman (according to the woman’s assessment)

Dependent variable: parameters of a woman’s interaction with her child	R^2^	Durbin–Watson statistic	ANOVA		Independent variables: basic need support	Coefficients		
			F	P		B	SE	β
Undemanding — **exactingness**	0.089	1.560	8.297	0.005	Constant	22.068	2.461	
					R_W_B_Now	–0.362	0.126	–0.298
So#ness — **strictness**	0.203	1.840	7.050	0.000	Constant	10.735	2.454	
					A_W_HM_Ch	–0.380	0.102	–0.385
					A_W_HM_Now (square)	0.012	0.004	0.295
					C_W_B_Now (square)	0.012	0.005	0.238
Autonomy — **ontrol**	0.125	1.935	12.140	0.001	Constant	24.033	2.578	
					A_W_HM_Now	–0.470	0.135	–0.354
Rejection — **acceptance**	0.317	2.151	7.522	0.000	Constant	35.529	16.054	
					R_W_B_Now (square)	0.151	0.057	4.807
					R_W_B_Now	–5.116	2.081	–4.527
					C_W_HM_Ch (square)	0.012	0.033	–4.345
					C_W_HM_Ch	3.685	1.112	4.146
					R_W_HM_Ch	–0.209	0.094	–0.281
Lack of cooperation — **cooperation**	0.473	2.371	14.564	0.000	Constant	–19.416	7.365	
					C_W_HM_Now	–0.676	0.127	–0.620
					A_W_HM_Now	0.636	0.153	0.454
					C_W_HM_Ch (square)	–0.170	0.027	–5.963
					C_W_HM_Ch	5.541	0.896	5.858
					A_W_B_Now	–0.211	0.097	–0.181
Inconsistency — **consistency of the parent**	0.196	1.859	10.267	0.000	Constant	–13.287	11.183	
					C_W_HM_Ch (square)	–0.143	0.042	–3.822
					C_W_HM_Ch	4.354	1.379	3.518
*Satisfaction with the relationship*	0.266	2.124	10.033	0.000	Constant	–3.170	7.304	
					R_W_B_Now (square)	0.008	0.003	0.286
					C_W_HM_Ch (square)	–0.098	0.028	–3.883
					C_W_HM_Ch	2.938	0.922	3.525

Note. B— non-standardized coefficient of the regression equation; SE is the standard error; β is the standardized coefficient of the regression equation; R2 is the coefficient of determination; F = Fisher’s F-test; p = significance. Critical probability = 5 %.

The results showed that there are linear associations between a woman’s basic need support in childhood by her mother and the woman’s basic need support of her own child. First, the more a woman’s mother supports her need for relatedness now, the more a woman supports her child’s need for autonomy. Second, the more a woman’s mother supported her need for competence in childhood, the more the woman supports the child’s need for relatedness now.

In addition to the linear ones, quadratic dependences were also found. According to the results, if the mother very highly supported, or very little supported, the woman’s need for competence in childhood, then the woman supports her child’s need for competence more in the present, and there was an asymmetry towards a positive relationship in this regard.

If the mother very highly supported, or very little supported, the woman’s need for relatedness in childhood, then in the present the woman supports the need for relatedness in her child less, and again there is an asymmetry towards a positive relationship. The combination of this quadratic relationship with a linear one means the asymmetry of the relationship is in the direction of a positive relationship.

If in the present the mother very highly supports, or very little supports, the woman’s need for competence, then the woman supports the need for relatedness in her child more, and here there was also an asymmetry towards a positive relationship.

Support for basic psychological needs in all three studied relationship systems became a statistically significant predictor for seven out of ten parameters of the “Parent-child interaction” questionnaire. At the same time, the percentage of the variance of the variables of the mother-child relationship explained by the support of these needs varied from 8.9 to 47.3. To the greatest extent, basic need support predicted such parameters of interaction as cooperation and acceptance, and did not at all predict emotional closeness, agreement, or parental authority.

A mother’s support for a woman’s need for competence in her childhood was a predictor of most (4 out of 7) parameters of a woman’s interaction with her child, namely, acceptance, cooperation, consistency of the parent, and satisfaction with the relationship. The support for the relatedness need that a woman received from her mother during childhood predicted the acceptance parameter, while the support she received during her childhood for autonomy predicted the parameter of strictness in her interaction with her own child.

The basic need support that a woman receives from her mother in the present was also a predictor of the mother-child interaction parameters. A mother’s support for the woman’s autonomy need in the present, predicted less control, more cooperation, and more or less strictness (nonlinear, U-shaped connection), while the mother’s support for the woman’s competence need in the present, predicted less cooperation in the mother-child interaction.

A woman’s basic need support for her child also predicted the parameters of the mother-child interaction. Supporting relatedness predicted acceptance (nonlinear, U-shaped connection), less exactingness, and greater satisfaction with the relationship; support for autonomy predicted less cooperation; and support for competence predicted strictness (nonlinear, U-shaped connection).

## Discussion

The picture of how a woman supports the basic needs for her child is similar to how those needs were supported by a woman’s mother in her childhood, and this confirms the first hypothesis: support for competence and relatedness were substantially the same in these intergenerational relationship systems, and both were significantly higher than support for autonomy in the respective system. Since similar results have been obtained using other methods and with a different population (Lynch, Salikhova, & Eremeeva, 2020), this pattern may characterize the attitude towards the child in Russian culture. Support for autonomy reaches the level of support for competence and relatedness only in the relationship of the mother to the adult woman.

Overall, we observed an increase in support for basic needs from generation to generation. We would like to think that this is due to the tendency of changes in Russian cultural standards towards greater support for the psychological needs of the child.

Results of the present study also supported the second hypothesis. What was most important for a woman, in order to support her child’s basic needs, was how her mother supported her competence and relatedness as a child. Interestingly, both extremely low and extremely high estimates of the support of a woman’s needs for competence and relatedness in her childhood by her mother, had a similar effect on the woman’s support of the corresponding needs of her own child. However, in the case of the support of a woman’s need for competence by her mother in childhood, both extremely low and extremely high scores predicted that she more strongly supported this need of her own child. On the other hand, in the case of the need for relatedness, both extremely low and extremely high scores predicted that in the relationship with her child, she would support this need less.

Another predictor of a woman’s basic need support for her child was how the mother supports her competence and relatedness now. The support of a woman’s need for relatedness by her mother in the present, was a predictor of a woman’s support for her child’s need for autonomy. At the same time, the mother’s support of the woman’s autonomy, whether in childhood or at present, did not play such a role, which distinguishes our results from those obtained in the Italian sample noted earlier (Costa et al., 2015).

The support of a woman’s competence by her mother in childhood turned out to be most widely associated with the various parameters of interaction of a woman with her child, and this confirms the third hypothesis. In general, the more that in a woman’s recollection, her mother in childhood supported her need for competence, the less acceptance, cooperation, consistency, and satisfaction in the relationship were present in the woman’s current relationship with her child. However, these associations were nonlinear, quadratic in nature: with extremely low values of competence support of a woman in childhood by her mother, smaller values of the same parameters of interaction between a woman and her child were observed.

To a large extent, these results correspond with the ideas developed in recent years by Grolnick, Raftery-Helmer, Marbell, Flamm, Cardemil, and Sanchez (2014), which emphasize that what is important is not only the very fact of support for a particular need of the child, but also how this support is carried out. In these studies, different means of supporting competence led to the child experiencing different degrees of their own autonomy (Grolnick et al., 2014), and different ways of controlling behavior, or, more precisely from the SDT perspective, differences in how mothers exercised authority, led to differences in the autonomous self-regulation and emotional state of the child (Levitt, Grolnick, Caruso, & Lerner, 2020). A more detailed study of the ways in which Russian women support their child’s need for competence may help to uncover more deeply the psychological mechanisms that lie behind our results.

It can be assumed that some means of supporting a person’s competence may be perceived as reflecting a highly evaluative attitude on the part of the parent. Alternatively, if a woman’s competence was very highly-supported in childhood, then, perhaps, she will be less ready to perceive her own child’s imperfection. This is probably why, in our data, there was less acceptance and cooperation with one’s child. On the whole, these results support Ryan and Deci’s suggestion (2017) that further research is needed to disentangle the various cross-cultural issues in providing basic need support.

It turned out that greater support for a woman’s need for autonomy during childhood was a predictor of her own less strictness with her child, which fully corresponds to an SDT perspective.

The present study showed that in the minds of Russian women, the highest levels of basic need support, with respect to the competence and autonomy needs in particular, were associated with negative consequences for a woman’s attitude towards, or interaction with, her child. From a self-determination theory perspective, it will be very important to disentangle this apparent paradox when optimizing child-rearing practices in ways that are cross-culturally appropriate.

## Conclusion

The social situation of development (to borrow Vygotsky’s concept) indeed demonstrated intergenerational continuity in such an important component as basic need support. In particular, support for the needs for relatedness and competence prevailed over support for the need for autonomy, both in the system of a woman’s relationship with her child and in the system of her relationship with her mother in childhood.

The main lines of continuity with respect to the child’s basic need support related to the needs for competence and relatedness, are that the more these needs were supported for the woman in her own childhood and were currently being supported by the woman’s mother, the more the woman supported them currently in her own child. At the same time, no intergenerational continuity was found in supporting the need for autonomy.

Basic need support in different intergenerational systems of relations was a predictor of most characteristics of a woman’s interaction with her child. These characteristics of the parent-child interaction were predominantly predicted by a woman having experienced support for competence by her own mother during childhood, by the mother supporting the woman’s needs for autonomy in the present, and by the woman supporting her own child’s need for relatedness in the present.

The present research can be used in the development of educational programs for expectant and current mothers, in particular with an emphasis on facilitating the woman’s psychological readiness to build effective interactions with her child while overcoming ineffective interactions; these goals can be addressed within the framework of family and parental counseling. Particular attention should be paid to the importance of supporting the child’s need for autonomy and the best, most effective means of supporting the need for competence, taking into account relevant cultural considerations.

## Limitations

The main limitation of this study is that only the responses of the woman herself were surveyed, and that she rated all three systems of relationships. It would be important to develop this research in the direction of interviewing her mother as well, and also through application of methods for observing the real interaction of a woman with her child.

The study is also limited by the sample size and the lack of control over some variability in the socio-demographic characteristics of the participants.
